# *In vitro* Characterization of Anti-SARS-CoV-2 Intravenous Immunoglobulins (IVIg) Produced From Plasma of Donors Immunized With the BNT162b2 Vaccine and Its Comparison With a Similar Formulation Produced From Plasma of COVID-19 Convalescent Donors

**DOI:** 10.3389/fmedt.2021.772275

**Published:** 2022-01-05

**Authors:** Gabriel Rojas-Jiménez, Daniela Solano, Álvaro Segura, Andrés Sánchez, Stephanie Chaves-Araya, María Herrera, Mariángela Vargas, Maykel Cerdas, Gerardo Calvo, Jonathan Alfaro, Sebastián Molina, Kimberly Bolaños, Andrés Moreira-Soto, Mauren Villalta, Adriana Sánchez, Daniel Cordero, Gina Durán, Gabriela Solano, Aarón Gómez, Andrés Hernández, Laura Sánchez, Marco Vargas, Jean Felix Drexler, Alberto Alape-Girón, Cecilia Díaz, Guillermo León

**Affiliations:** ^1^Sección de Virología Médica, Departamento de Microbiología e Inmunología, Facultad de Microbiología, Universidad de Costa Rica, San José, Costa Rica; ^2^Instituto Clodomiro Picado, Factulad de Microbiología, Universidad de Costa Rica, San José, Costa Rica; ^3^Laboratorio Clínico y Banco de Sangre de la Universidad de Costa Rica, Oficina de Bienestar y Salud, Universidad de Costa Rica, San José, Costa Rica; ^4^Banco Nacional de Sangre, Gerencia Médica, Caja Costarricense del Seguro Social, San José, Costa Rica; ^5^Institute of Virology, Charité-Universitätsmedizin Berlin, Corporate Member of Freie Universität Berlin, Humboldt-Universität zu Berlin, and Berlin Institute of Health, Berlin, Germany; ^6^Centro de Investigación en Enfermedades Tropicales (CIET), Facultad de Microbiología, Universidad de Costa Rica, San Jose, Costa Rica; ^7^German Centre for Infection Research (DZIF), Associated Partner Charité-Universitätsmedizin Berlin, Berlin, Germany; ^8^Departamento de Bioquímica, Escuela de Medicina, Universidad de Costa Rica, San José, Costa Rica

**Keywords:** BNT162b2 vaccine, convalescent plasma, COVID-19, hyperimmune plasma, hyperimmune polyclonal antibodies, IVIg, passive immunotherapy, SARS-CoV-2

## Abstract

Despite vaccines are the main strategy to control the ongoing global COVID-19 pandemic, their effectiveness could not be enough for individuals with immunosuppression. In these cases, as well as in patients with moderate/severe COVID-19, passive immunization with anti-SARS-CoV-2 immunoglobulins could be a therapeutic alternative. We used caprylic acid precipitation to prepare a pilot-scale batch of anti-SARS-CoV-2 intravenous immunoglobulins (IVIg) from plasma of donors immunized with the BNT162b2 (Pfizer-BioNTech) anti-COVID-19 vaccine (VP-IVIg) and compared their *in vitro* efficacy and safety with those of a similar formulation produced from plasma of COVID-19 convalescent donors (CP-IVIg). Both formulations showed immunological, physicochemical, biochemical, and microbiological characteristics that meet the specifications of IVIg formulations. Moreover, the concentration of anti-RBD and ACE2-RBD neutralizing antibodies was higher in VP-IVIg than in CP-IVIg. In concordance, plaque reduction neutralization tests showed inhibitory concentrations of 0.03–0.09 g/L in VP-IVIg and of 0.06–0.13 in CP-IVIg. Thus, VP-IVIg has *in vitro* efficacy and safety profiles that justify their evaluation as therapeutic alternative for clinical cases of COVID-19. Precipitation with caprylic acid could be a simple, feasible, and affordable alternative to produce formulations of anti-SARS-CoV-2 IVIg to be used therapeutically or prophylactically to confront the COVID-19 pandemic in middle and low-income countries.

## Introduction

SARS-CoV-2 (Severe Acute Respiratory Syndrome Coronavirus 2) is an enveloped, positive sense RNA virus, composed of four structural proteins: envelope (E), membrane (M), nucleocapsid (N), and the homotrimeric spike (S) protein. Each S protein monomer consists of S1 and S2 subunits, and possesses several functional domains (1), including the receptor binding domain (RBD) located in the S1 subunit (2).

The virion infects human cells, as well as some other mammalian and avian cells, through the interaction between RBD and the angiotensin-converting enzyme 2 (ACE2) (1). In most cases, infection by SARS-CoV-2 is asymptomatic. Occasionally, however, after an incubation period of 2–14 days, patients develop the coronavirus disease 2019 (COVID-19) (3).

Signs and symptoms of COVID-19 range from mild/moderate (dry cough, fever, tiredness, chills, sore throat, loss of smell and taste, headache, body pain, nasal congestion, diarrhea, nausea, and vomiting) to severe (thrombosis, cardiac damage, and massive alveolar damage, respiratory failure, and death) (3). Severe cases are more frequent in elderly patients with comorbidities, such as high blood pressure, heart and lung diseases, diabetes, and cancer (3).

After the first cases reported in Wuhan, COVID-19 spread rapidly throughout the world, acquiring the status of pandemic, declared by the World Health Organization (WHO), in March 2020. By the end of November 2021, more than 257 million cases, with more than 5.1 million deaths, had been reported worldwide. To reduce the progress of the pandemic, the WHO has recommended precautionary measures, such as the use of masks, hand washing, social distancing, and lockdowns. Nevertheless, the pandemic has not ceased, and it seems there is still much to do to control it.

Despite the large number of investigations carried out so far, COVID-19 disease mechanism/pathophysiology is largely unknown. In cases requiring hospitalization, support therapy consists mainly of oxygenation, fluid management and ventilation (3). Given the absence of effective treatments for treating the disease, many efforts to reduce the effects of COVID-19 have focused on the development of vaccines and immunoglobulin formulations, for active and passive immunization, respectively (4, 5).

In both cases, anti-RBD antibodies have been identified as promising candidates to increase resistance against infection (1, 6, 7). Aside from the prevention of the ACE2-RBD interaction, those antibodies could trigger the complement system and activate mononuclear cells via Fc receptors (4, 8), thus contributing to limit the course of the disease.

Several anti-COVID-19 vaccines have been developed (5), and some of them are being produced at industrial scale and distributed mainly to high-income countries (9). Although the demonstration of effectiveness and immunity duration induced by these vaccines is still a work in progress (10), vaccination is emerging as the main strategy to control the pandemic (11).

Despite the potential of anti-COVID-19 vaccines as prophylactic drugs, their effectiveness could be diminished in individuals with lymphopenia, primary/secondary antibody deficiencies, or suffering from moderate/severe COVID-19. In these cases, passive immunization with preparations of anti-SARS-CoV-2 antibodies might be a therapeutic option (8).

Examples of this type of preparations are: (1) convalescent plasma (i.e., plasma obtained from patients who recovered from COVID-19) (8), (2) vaccinated plasma (i.e., plasma obtained from individuals immunized with anti-COVID-19 vaccines) (8), (3) intravenous immunoglobulins (IVIg) purified from convalescent plasma (i.e., CP-IVIg) (12, 13), (4) IVIg produced from plasma of vaccinated donors (i.e., VP-IVIg), (5) monoclonal antibodies (14), and (6) animal-derived immunoglobulins (15).

In this work, we prepared a VP-IVIg from plasma of donors immunized with the BNT162b2 (Pfizer-BioNTech) anti-COVID-19 vaccine and compared its *in vitro* efficacy and safety with those of a similar CP-IVIg formulation. Moreover, we evaluated the performance of the caprylic acid precipitation method at pilot-scale as a plasma fractionation downstream strategy to produce both formulations.

## Materials and Methods

### Ethics Statement

Collection and use of human plasma were approved by the Central Committee of Pharmacotherapy (Act GM- CCF-1854−2020) and the Institutional Bioethics Committee of *Caja Costarricense de Seguro Social* (C.C.S.S.; Costa Rican Social Security Fund). All methods were carried out following the regulations emitted by C.C.S.S., the Costa Rican Ministry of Health and the University of Costa Rica. All donors were over 18 years old and provided informed consent.

### Vaccinated and Convalescent Plasma

Vaccinated plasma was obtained by *Banco de Sangre de la Universidad de Costa Rica* from the blood of 101 donors who had been immunized with the BNT162b2 anti-COVID-19 vaccine (Pfizer-BioNTech), within the first 12 weeks after vaccination. Convalescent plasma was obtained by C.C.S.S. through of the *Banco Nacional de Sangre*, from the blood of 158 donors who were diagnosed by direct detection of SARS-CoV-2 RNA in swabs of the upper respiratory tract, developed mild or moderate symptomatology of COVID-19, and made a full recovery. Collection of convalescent plasma was performed within the first 12 weeks after convalescence. In both cases, donors meet common requirements for blood donation, including they were 18–65 years old, 1.5 m tall and more than 50 Kg bodyweight. All donors were negative for HBV, HCV and HIV. Pregnant women or people with epilepsy, syphilis, malaria, Chagas disease, cancer, or serious heart disease were excluded. There were no concerns regarding ABO or Rh blood type. Blood was collected by venipuncture of the median cubital vein, with a needle (16–17 gauge) coupled to a system of polyvinyl chloride (PVC) bags, with citrate-dextrose (ACD) as anticoagulant. Blood bags were centrifuged during 10 min at a relative centrifugal force (RCF) of 5,000 xg and 24°C. Supernatant plasma was separated with a manual plasma extractor and stored at −20°C until use.

### Screening of Plasma Units

Anti-SARS-CoV-2 RBD (anti-RBD) antibodies, ACE2-RBD neutralizing antibodies, and total IgG, IgM, and IgA were determined by chemiluminescent immunoassay (CLIA) using a MAGLUMI 600TM equipment (Snibe Diagnostic) and the following kits: SARS-CoV-2 S-RBD IgG (REF 130219017M), ACE2-RBD Neutralizing Antibodies (REF 130219027M), IgG serum analysis (REF 130608005M), IgM (REF 130608002M), and IgA serum analysis (REF 130608003M), respectively. ID-NAT screening was conducted using the Procleix Ultrio Elite (UE) multiplex assay for the detection of HBV, HCV and HIV, on the Panther platform (Grifols Diagnostic Solutions Inc.).

### Pilot-Scale Production of VP-IVIg and CP-IVIg

**Figure 1:**
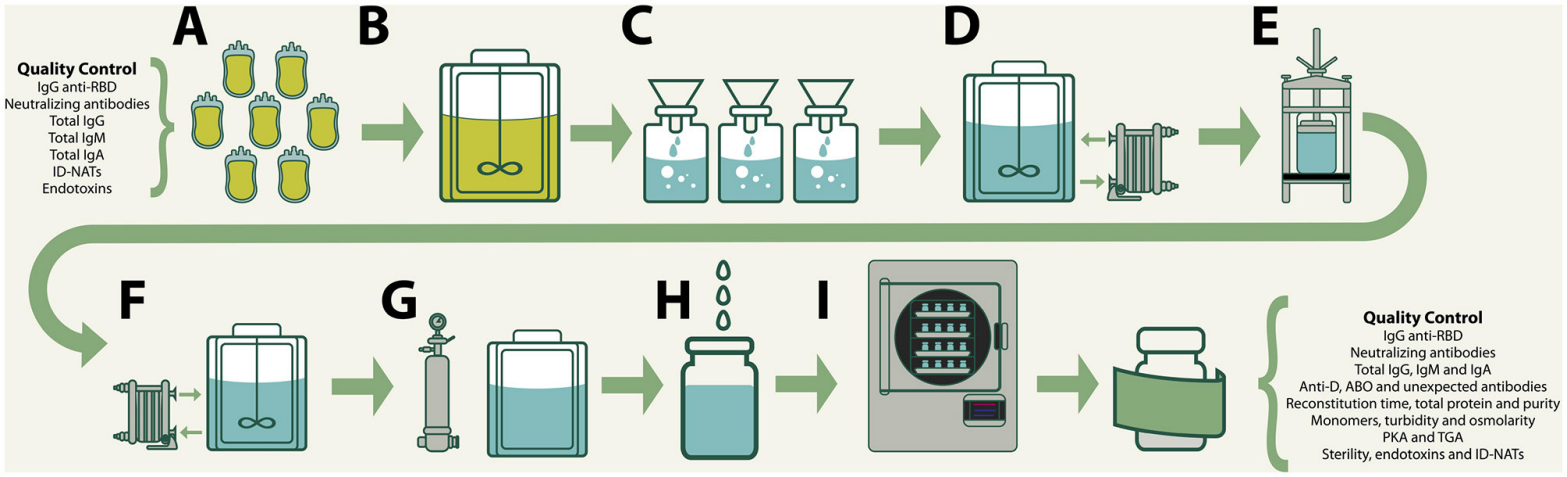
Production of VP-IVIg and CP-IVIg by the method of caprylic acid precipitation: **(A)** Collection, testing and approval of raw plasma. **(B)** Precipitation of plasma proteins by addition of caprylic acid 5–6% (v/v). **(C)** Removal of precipitates by gravity filtration. **(D)** Diafiltration, concentration and formulation. **(E)** Strong anion exchanger chromatography. **(F)** Readjustment of the formula. **(G)** Sterilizing filtration. **(H)** Filling in type 1 borosilicate vials (40 mL/vial). **(I)** Lyophilization.

VP-IVIg and CP-IVIg were produced by the method of caprylic acid precipitation at room temperature (16, 17). In brief, undiluted pooled plasma (from either vaccinated or convalescent donors) was vigorously stirred, while caprylic acid was slowly added, at physiological pH, to attain a concentration of 5–6% (v/v). Stirring was maintained for 60 min. Then, the precipitate was removed by gravity filtration through a Whatman Grade 2V qualitative filter paper (Cat: 1202-500). The filtrates were diafiltered, concentrated and formulated at 5–7.0 g/dL total protein concentration, 9 g/L NaCl and pH 7.0. In order to remove traces of albumin, ceruloplasmin and other unwanted contaminants that remained after caprylic acid precipitation, the formulations were processed with 1 L of a strong anion exchanger (Ceramic HyperD, Pall) in a glass chromatography column (BPG 100, GE), using 150 mM NaCl, pH 7.0 as eluent, with a 1,000 mL/min flow rate. After re-adjusting at 5–7.0 g/dL total protein concentration, 9 g/L NaCl and pH 7.0, the formulations were sterilized by filtration, dispensed in type 1 borosilicate vials (40 mL/vial) and stabilized by lyophilization. A schematic summary of the process is presented in Figure 1.

### *In vitro* Efficacy of the Anti-SARS-CoV-2 IVIg Formulations

Anti-RBD and ACE2-RBD neutralizing antibodies were determined by CLIA, as described above. Previously, an elevated correlation between the concentration of these antibodies and the plaque reduction neutralization test (PRNT) has been demonstrated (18). In order to test this, PRNT were performed as previously described (19), using the SARS-CoV-2 isolate (Germany/Gisaid_EPI_ISL_406862) at inhibitory concentrations ranging from 0.558–0.004 g/L for VP-IVIg and 0.692–0.005 g/L for CP-IVIg.

### *In vitro* Safety of the Anti-SARS-CoV-2 IVIg Formulations

#### Immunological Evaluation

Total IgG, IgM and IgA were determined by CLIA, as described above. Screening of anti-D, anti-A, anti-B and irregular antibodies was performed by the column agglutination method: anti-D, anti-A and anti-B antibodies were studied with Serigrup Diana A_1_/B cells in DG Gel Neutral cards (Grifols Diagnostic Solutions Inc.), while irregular antibodies were studied with Serascan Diana 3/3P cells in DG Gel 8 Anti-IgG (Rabbit) cards (Grifols Diagnostic Solutions Inc.).

#### Physicochemical Evaluation

Total protein concentration was determined by the Biuret method (20). Purity was determined by zone electrophoresis on cellulose polyacetate followed by a densitometric analysis. The percentage of monomers was determined by HPLC (Agilent 1100 series: Agilent Technologies) in an Agilent Bio SEC-3 300ª column (7.8 × 300 mm), using 150 mM phosphate buffer (pH 7.0) as eluent, with a 1.0 mL/min flow rate and detection at 214 nm. Turbidity was determined using a turbidimeter (La Motte, model 2020), and expressed as nephelometric turbidity units (NTU). Osmolality was determined with a micro-osmometer (Advanced™ MicroOsmometer, model 3300, Advanced Instruments, Inc.).

#### Biochemical Evaluation

Prekallikrein activator (PKA) amidolytic activity was determined with a PreKallikrein Activator Assay Kit (S-2302; Pathway Diagnostics). The thrombin generation assay (TGA) was performed with the Technothrombin TG kit (Technoclone), the RC High reagent, and the samples diluted at 1 g/dL in fresh platelet poor plasma.

#### Microbiological Evaluation

Sterility and endotoxin content were assessed according to the United States Pharmacopeia 42/NF 37 (21, 22). Endotoxin content was determined by the gel clot method of the *Limulus* Amebocyte Lysate (LAL) assay. Briefly, 0.2 mL of the formulation were added to a single test vial of LAL reagent (Pyrotell® Associates of Cape Cod Incorporated, cat #65003). Then, the vials were incubated at 37 ± 1°C for 60 ± 2 min. After, the tubes were gently inverted 180° to assess gelification of the mixture. LPS standard (Associates of Cape Cod Incorporated, ACC cat # E0005) diluted in LAL Reagent Water (ACC, cat # WP1001) was used as positive control. LAL Reagent Water was used as a negative control. Endotoxin limit of anti-SARS-CoV-2 IVIg (i.e., 4.2 EU/mL) was calculated as the quotient of the threshold pyrogenic dose of endotoxin per kilogram of body weight (i.e., 5 EU/kg), divided by the maximum total dose administered to a 100 kg-patient for 1 h (i.e., 120 mL; USP, 2014b).

### Evaluation of the Yield of the Pilot-Scale Fractionation Method

The recovery of the fractionation method was assessed as the percentage of the total amount of anti-RBD antibodies in the raw plasma retrieved in the bulk of each formulation. Also, the yield was determined as the number of 40-mL vials obtained in each batch per volume of the corresponding raw plasma.

### Statistical Analysis

Differences in concentration of anti-RBD antibodies between vaccinated and convalescent plasma were tested by one-way ANOVA, considering the assumptions of linearity and homogeneity of variances. Differences in the concentration of anti-RBD antibodies at several times after vaccination were tested by a general linear model of repeated measures. The assumption of sphericity was tested by Mauchly's test of Sphericity, and any deviation was corrected by using the Greenhouse-Geisser factor. Values of *p* < 0.05 were considered statistically significant. The tests were conducted using the software IBM SPSS v. 25.0. The inhibitory concentration (IC_50_) in PRNT was calculated using a non-linear regression analysis in the GraphPadPrism 5 software.

## Results and Discussion

### Characterization of Vaccinated and Convalescent Plasma

**Figure 2:**
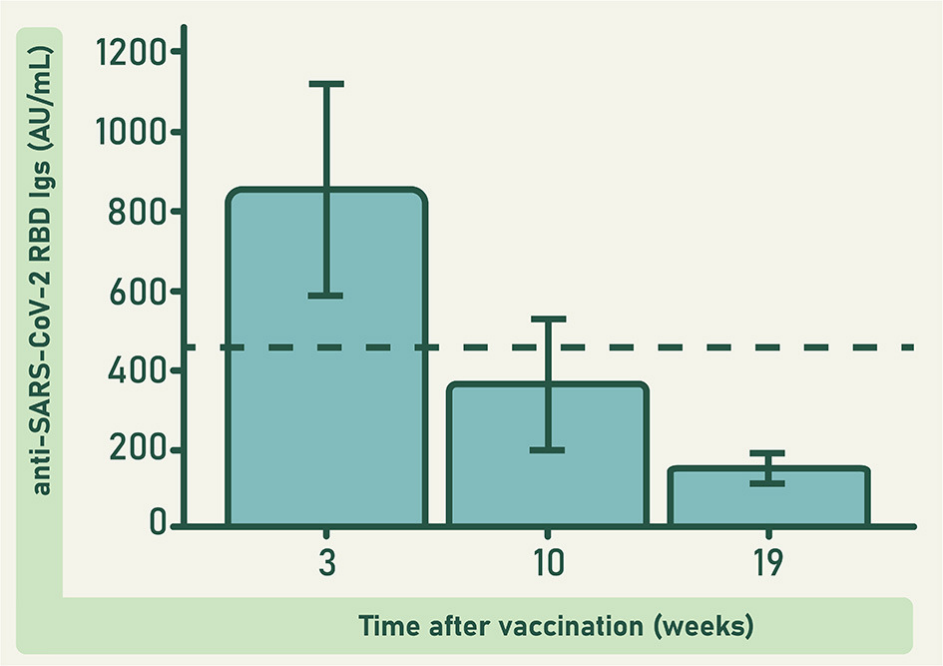
Concentration of anti-RBD antibodies in the plasma of 23 donors immunized with the BNT162b2 anti-COVID-19 vaccine, at different weeks after vaccination. The dashed line represents the observed grand mean. Bars represent the estimated marginal means ± 95 CI. Differences at different times were significant (*F* = 35.560; d*f* = 2; 40; *p* ≤ 0.0001).

Vaccinated donors in this study were immunized with two doses of the BNT162b2 anti-COVID-19 vaccine (Pfizer-BioNTech), separated by a 3-week lapse. Concentration of anti-RBD antibodies was determined in 23 of these donors at 3, 10, and 19 weeks after the second dose. None of these donors had a known history of COVID-19. Findings revealed a rapid increase in anti-RBD antibody concentration, followed by a gradual but significant decrease in the subsequent weeks (*F* = 35.560; d*f* = 2; 40; *p* ≤ 0.0001; Figure 2). This result agrees with previous reports (23). Similarly, the response induced by SARS-CoV-2 infection follows a pattern characterized by an initial rapid increase in the serum concentration of anti-SARS-CoV-2 antibodies, that around 3 weeks after the first symptoms reaches a peak, and then gradually decreases (24–26).

**Figure 3:**
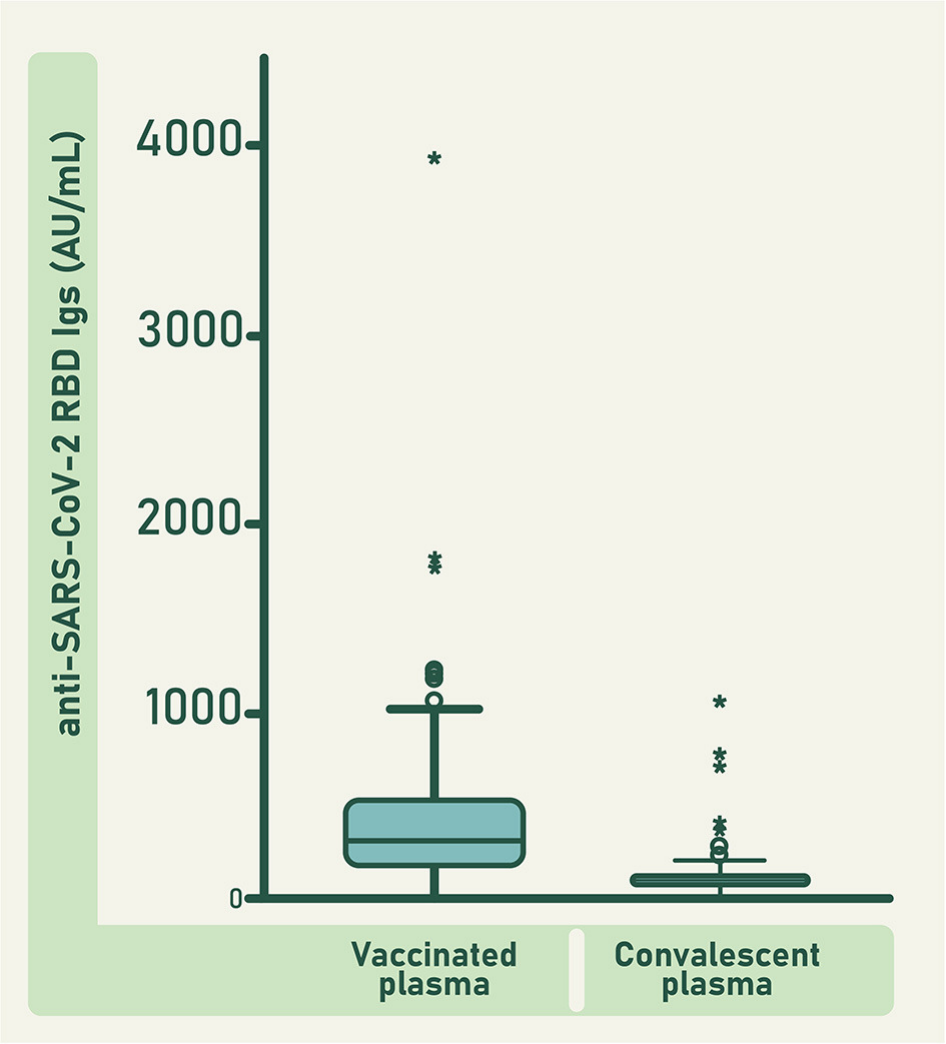
Concentration of anti-RBD antibodies in individual units of vaccinated and convalescent plasma. Bars represent the mean value ± 95 CI. Circles represent the outlier data points and asterisks represent the extreme outlier data points. Difference between both groups was significant (*F* = 57.435; d*f* = 1; 301; *p* ≤ 0.0001).

When comparing anti-RBD antibodies concentration between vaccinated and convalescent donors (Figure 3), the variability observed in each group is a result of several issues that include the differences in the individual characteristics of the immune system of all donors, and the severity of the disease experimented by the convalescent donors. Vaccinated and convalescent plasma were collected within the first 12 weeks after vaccination or convalescence, respectively.

**Figure 4:**
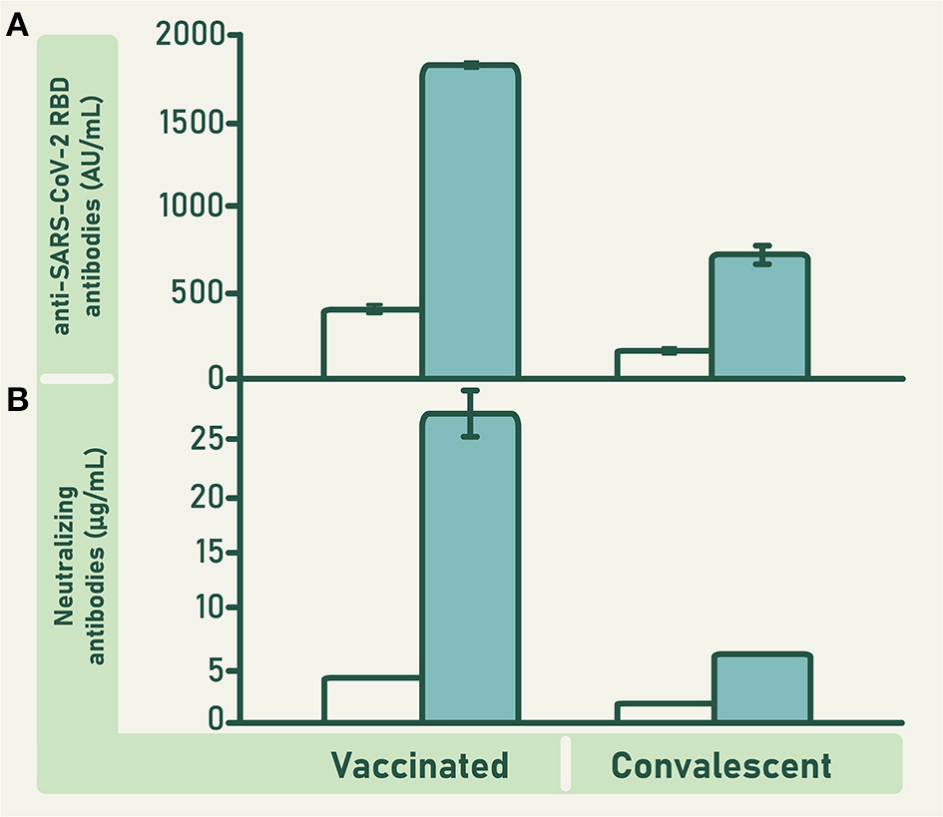
Concentration of anti-RBD antibodies **(A)** and ACE2-RBD neutralizing antibodies **(B)** in the pool of plasma collected from vaccinated and convalescent donors (empty bars), and in the VP-IVIg and CP-IVIg (full bars). Bars represent means ± SD of analyzes performed in triplicate. The concentration of anti-RBD antibodies in VP-IVIg and CP-IVIg corresponds to 7902 ± 69 and 3109 ± 199 binding antibody units (BAU)/mL, respectively.

The mean concentration of anti-RBD antibodies in the units of vaccinated plasma was significantly higher than that of the units of convalescent plasma (*F* = 57.435; d*f* = 1; 301; *p* ≤ 0.0001; Figure 3). Consequently, the concentration of anti-RBD and ACE2-RBD neutralizing antibodies in the pool of raw vaccinated plasma was higher than in the pool of raw convalescent plasma (Figure 4). Both, vaccinated and convalescent plasma had normal values of IgG, IgM and IgA, and all individual units were negative for HBV, HCV and HIV (Table 1).

**Table 1:** Characterization of anti-SARS-CoV-2 IVIg formulations^*^.

	**Type of plasma**			
	**Vaccinated**	**Convalescent**	Comercial Controla^**^	
Plasma	Batch ID	6700621DECHLF	6650521DECHLF	CSL4340200191
	IgG (g/L)	8.9 ± 0.3	10.8 ± 0.4	–
	IgM (g/L)	1.2 ± 0.0	1.4 ± 0.0	–
	IgA (g/L)	1.8 ± 0.1	2.0 ± 0.1	–
	ID-NAT-HIV	Negative	Negative	–
	ID-NAT-HCV	Negative	Negative	–
	ID-NAT-HBV	Negative	Negative	–
Final formulation	IgG (g/L)	42.9 ± 3.	52.8 ± 3.3	61.3 ± 1.9
	IgM (g/L)	1.1 ± 0.1	3.8 ± 1.1	0.1 ± 0.0
	IgA (g/L)	6.6 ± 0.4	7.0 ± 0.1	0.3 ± 0.0
	Anti-D antibodies	Negative	Negative	Negative
	Anti-A antibodies	1/16	1/32	1/1
	Anti-B antibodies	1/16	1/8	Negative
	Irregular antibodies	Negative	Negative	Negative
	pH	7.0	7.0	6.5
	Total protein (g/L)	55.8 ± 0.3	69.2 ± 0.2	58.6 ± 0.1
	Purity (%)	84	86	100
	Monomers (%)	90.0%	88.3%	93.0 %
	Turbidity (NTU)	23.3 ± 0.6	28.6 ± 0.8	66.3 ± 5.5
	Osmolality (mOsm/kg)	233.0 ± 1.0	228.0 ± 1.0	456.8 ± 2.9
	PKA (IU/mL)	2.4 ± 0.0	<1.56	<1.56
	TGA thrombin (nM)	144.5 ± 0.6	150.5 ± 26.4	139.6 ± 19.9
	Sterility	No growth	No growth	No growth
	Endotoxin (EU/mL)	<4.2 EU/mL	<4.2 EU/mL	<4.2 EU/mL
	ID-NAT-HIV	Negative	Negative	Negative
	ID-NAT-HCV	Negative	Negative	Negative
	ID-NAT-HBV	Negative	Negative	Negative
Process	Amount of plasma units	101	158	–
	Plasma volume (L)	20	32	–
	Number of 40-mL vials	39	48	–
	Recovery (%)	35	27	–
	Yield (vials/L of plasma)	2.0	1.5	–

* *Results are presented as the average ± SD of a triplicate of determinations.*

** *The commercial control is used as reference*.

### *In vitro* Efficacy of VP-IVIg and CP-IVIg

Some clinical evidence suggests that convalescent plasma transfusion may not be able to reduce the mortality or ventilation requirement in hospitalized patients; hence, the effectiveness of this immunotherapy remains controversial (8, 10, 27). However, a CP-IVIg formulation obtained by the caprylic acid method was tested in a clinical trial in Pakistan, showing that its administration in severe and critical COVID-19 patients is safe, increases the possibility of survival, and reduces the risk of disease progression (28).

Anti-SARS-CoV-2 antibodies in CP-IVIg include antibodies toward S and N proteins. In contrast, anti-SARS-CoV-2 antibodies in VP-IVIg are exclusively directed against RBD. Although this could be interpreted as an advantage in favor of CP-IVIg, there is evidence showing that this difference in the repertoire of specificities does not translate into differences in the *in vitro* neutralizing ability of both formulations (29). This result suggests that most of the ACE2-RBD neutralizing antibodies are anti-RBD antibodies.

**Figure 5:**
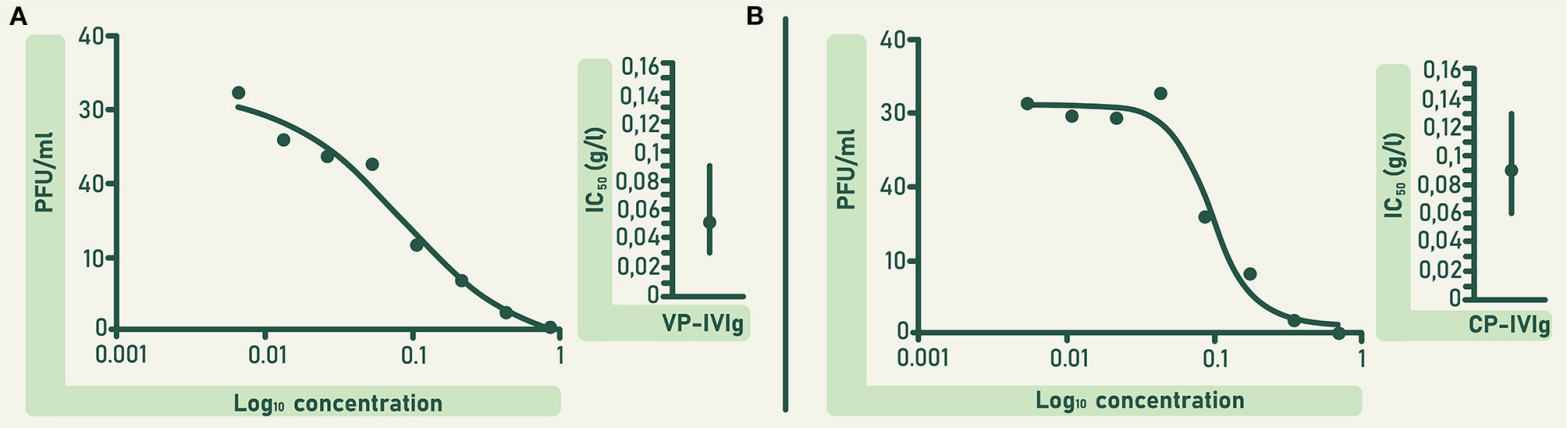
*In vitro* neutralizing potency of VP-IVIg **(A)** and CP-IVIg **(B)** determined by PRNT. Dose-response curves are shown left and mean inhibitory concentrations of both formulations are shown right. IC_50_ was calculated using a non-linear regression analysis in the GraphPadPrism 5 software. Vertical solid lines denote 95% confidence intervals for both formulations.

The concentration of anti-RBD and ACE2-RBD neutralizing antibodies in CLIA were higher in VP-IVIg and CP-IVIg than in their respective raw plasma (Figure 4). Moreover, the concentration of these antibodies was higher in VP-IVIg than in CP-IVIg (Figure 4). The concentration of anti-RBD antibodies in VP-IVIg and CP-IVIg corresponds to 7902 ± 69 and 3109 ± 199 binding antibody units (BAU)/mL, respectively (30). Results of ACE2-RBD neutralizing antibodies in CLIA were further confirmed using a PRNT (Figure 5), where the observed IC_50_ concentrations were lower for the VP-IVIg (mean: 0.05 g/L; 95% confidence intervals (CI):0.03–0.09) than for the CP-IVIg (mean: 0.09 g/L; 95% CI: 0.06–0.13). Therefore, it could be hypothesized that the efficacy of VP-IVIg would be slightly higher than that of CP-IVIg in a clinical setting.

We also measured the concentration of ACE2-RBD neutralizing antibodies in two equine hyperimmune formulations purified from plasma of horses immunized with recombinant S1 or a mixture of recombinant S1, N and a S-E-M mosaic (29). The results were 1198 ± 84 and 1074 ± 33 μg/mL, respectively. The concentration of ACE2-RBD neutralizing antibodies in a control formulation of equine immunoglobulins was <0.3 μg/mL (i.e., negative).

Despite the concentration of ACE2-RBD neutralizing antibodies in VP-IVIg is around 40 times lower than in the equine-derived formulations, human-derived formulations do not have pharmacokinetic limitations due to the foreignness of heterologous formulations (31). A longer half-life of the human whole IgG compared to the equine whole IgG is expected. In this sense, the comparison of the clinical efficacy between human and animal-derived anti-SARS-CoV-2 formulations remains to be studied.

### *In vitro* Safety of VP-IVIg and CP-IVIg

Both VP-IVIg and CP-IVIg were composed mainly of IgG (Table 1). The titer of anti-D antibodies in both formulations was lower than the pharmacopeial specification defined for this type of products (i.e., 1/64; Table 1), consequently, hemolytic reactions mediated by these antibodies are unlike. Moreover, despite the presence of IgM immunoglobulins (Table 1), the concentrations of ABO system antibodies were lower than the requirements defined for this kind of products (i.e., 1/64; Table 1). This result is produced by the combination of several factors including the initial concentration of anti-A and anti-B antibodies in the raw plasma, the dilution of anti-A and anti-B antibodies in the various plasma types on the pool, and a slight reduction of IgM after caprylic acid precipitation (Table 1). Thus, in contrast to vaccinated or convalescent plasma, administration of VP-IVIg or CP-IVIg will not require donor blood type match.

Since both VP-IVIg and CP-IVIg have residual IgA (Table 1), their administration to IgA-deficient patients may be either avoided or patients must consider a higher probability of occurrence of adverse reactions, in number and severity, compared to the normal population (32). In addition, both IVIg formulations were negative for irregular antibodies (Table 1). Presence or absence of irregular antibodies in the formulations is consequence of the concentration of these antibodies in the raw plasma and their dilution in the entire pool. It is not related to the fractionation process.

Purity of VP-IVIg and CP-IVIg was lower than 95% and requires to be improved (Table 1). But total protein concentration, IgG monomer percentage, turbidity and osmolality comply with customary values for IVIg formulations (Table 1). Both formulations had PKA activity lower than 35 IU/mL (i.e., the specification of *in vitro* hypotensive risk for this type of formulations) and TGA thrombin concentration lower than 350 nM (i.e., the specification of *in vitro* thrombogenic activity) (33). Additionally, VP-IVIg and CP-IVIg fulfilled sterility and endotoxin tests requirements. As expected, both formulations were negative for HBV, HCV and HIV (Table 1).

There is a theoretical risk that VP-IVIg and CP-IVIg could produce antibody-dependent enhancement (ADE) of the infection by SARS-CoV-2, but clinical evidence does not support this hypothesis (34). Some researchers have found a correlation between the administration of anti-SARS-CoV-2 antibodies and clearance of viral load. Also, VP-IVIg and CP-IVIg have a lower risk to induce adverse reactions than animal-derived formulations (35). Based on its *in vitro* safety profile, VP-IVIg could be suitable as a therapeutic alternative for clinical cases of COVID-19.

### Performance of the Caprylic Acid Method Fractionation at Pilot-Scale

The caprylic acid precipitation is a well stablished method for immunoglobulin purification. Compared with the traditional Cohn method (i.e., cold ethanol precipitation), the caprylic acid method is simpler, cheaper, more productive and has fewer specialized equipment requirements (16, 17). However, owing albumin and other therapeutic plasma proteins are denatured during precipitation, its productivity is limited to immunoglobulins formulations. Previous purification steps must be introduced before caprylic acid precipitation, to obtain formulations other than IVIg. Moreover, supplementary polishing and antiviral steps are required to meet the current specifications of purity and safety for IVIg.

Twenty-seven percent of the anti-RBD antibodies present in the staring pool of convalescent plasma was recovered in the bulk batch of CP-IVIg, to yield 1.5 vial/L of plasma (Table 1). On the other hand, 35% of the anti-RBD antibodies present in the pool of raw vaccinated plasma was recovered in the bulk batch of VP-IVIg, to yield 2.0 vials/L of plasma (Table 1). Similar recovery and yield between CP-IVIg and VP-IVIg was not surprising, since both formulations were produced by the same method. The reason why we obtained a lower recovery than the theoretically expected for the caprylic acid method (i.e., 60–70%), is that a loss of IgG was experienced due to the high dead volume in the pipe transfer lines, valves and downstream-processing equipment of our pilot-scale process line, instead of IgG denaturation along the process.

## Final Remarks

Convalescent plasma transfusion has been used worldwide during the current pandemic in the therapy of COVID-19 patients. When administered promptly convalescent plasma contributes to viral clearance and improves patient survival. However, in some developing countries convalescent plasma transfusion may heighten the risk of transmission of blood-borne pathogens. The plasma fractionation technology applied in this work to produce IVIg includes one viral removal/inactivation step. Therefore, it provides a feasible option to manufacture a therapeutic for COVID-19 which is safer than the use of convalescent plasma.

VP-IVIg and CP-IVIg have immunological, physicochemical, biochemical, and microbiological characteristics that meet the specifications of IVIg formulations. Therefore, both formulations could be candidates to be tested in clinical trials in terms of their efficacy and safety, in patients with lymphopenia, primary/secondary antibody deficiencies, or suffering from moderate/severe disease. Owing availability of vaccinated plasma is likely to increase with the advancement of vaccination campaigns, production of VP-IVIg could be easily scalable by using a simple method such as caprylic acid precipitation.

## Data Availability Statement

The original contributions presented in the study are included in the article, further inquiries can be directed to the corresponding author/s.

## Ethics Statement

The studies involving human participants were reviewed and approved by Institutional Bioethics Committee of Caja Costarricense de Seguro Social (C.C.S.S.; Costa Rican Social Security Fund). The patients/participants provided their written informed consent to participate in this study.

## Author Contributions

GL, CD, and AA-G designed the study and wrote the paper. GR-J, DS, ÁS, AnS, SC-A, MH, MVa, MC, GC, JA, SM, KB, AM-S, MVi, AdS, DC, GD, GS, AG, AH, LS, MVa, and JD performed experiments and analyzed the data. All authors have read and agreed to the published version of the manuscript.

## Funding

This study was supported by Banco Centroamericano de Integración Económica (BCIE; Resolution No. DI-87/2020), Ministerio de Ciencia, Innovación, Tecnología y Telecomunicaciones de Costa Rica (MICITT; Project FV-0001-20), the Vicerrectoría de Investigación, Universidad de Costa Rica (Project 741-C0-198), and the Global Centres for Health and Pandemic Prevention from the German academic exchange services (DAAD) (Grant Agreement: 57592642).

## Conflict of Interest

The authors declare that the research was conducted in the absence of any commercial or financial relationships that could be construed as a potential conflict of interest.

## Publisher's Note

All claims expressed in this article are solely those of the authors and do not necessarily represent those of their affiliated organizations, or those of the publisher, the editors and the reviewers. Any product that may be evaluated in this article, or claim that may be made by its manufacturer, is not guaranteed or endorsed by the publisher.
